# Paraffin Noses

**Published:** 1903-01-24

**Authors:** 


### PARAFFIN NOSES.

Mr. Stephen Paget has been showing a series of paraffin noses to the post-graduate students at the West London Hospital, and certainly in some of the cases the æsthetic effect was admirable. He summed up his estimate of what the injection of paraffin will do by saying that the general results are very good. A pure Greek profile cannot of course be promised, but one can promise a fairly good one, and one that shall at least be straight enough to be unnoticeable. The skin between the eyes can be so raised and moulded as to make this part quite good looking, even though for a quarter of a century or more it has been absolutely flat and ape-like. Nor is the improvement confined to the contour of the nose. When the portion between the eyes is raised, the appearance of the eyes themselves is improved, and the whole face is made to look more intellectual. Moreover, when the middle portion of the nose is raised, the organ becomes slightly lengthened, and thus it is possible to slightly depress the tip so that the nostrils lose their forward open aspect. The paraffin which Mr. Stephen Paget recommends is that of Pfannenstiel, which melts at a temperature of 115° F. This is easy to inject, easy to mould, not too hot, and sets perfectly hard. Lately he has been using a mixture of two paraffins which melts at 111° F., and he thinks this also is excellent. He is distinctly adverse to the use of Eckstein's paraffin, which melts at the very high temperature of 136° F. This is difficult to manipulate, it sets very rapidly and causes much swelling and some inflammation. Eckstein's, however, is the best form of syringe. But its needle is too long and not rigid enough, which, however, can be altered. It is an interesting question what happens ultimately to the paraffin. So far as he can gather from the literature on the subject, Mr. Paget believes that the hard Eckstein's paraffin becomes encapsuled, while those forms which melt at a point from 104° to 110° or 115° F., are very slowly replaced by fibrous tissue. For the details of the operation, reference may be made to the *British Medical Journal*, January 3, 1903. It hardly need be said that the feasibility of giving rigidity to the tissues and altering their form by the injection of paraffin having once been demonstrated, there are many possible applications for the process besides the making of noses.

